# Flow Training Program: Mindfulness, Decision Making, and Mental Well-Being of Young and Adult Elite Handball Athletes

**DOI:** 10.3390/sports12060160

**Published:** 2024-06-07

**Authors:** Luis Martiny, Gonçalo Dias, José Pedro Ferreira, Rodrigo Mendes, Rui Mendes

**Affiliations:** 1 Instituto Federal do Rio Grande do Norte, Natal 59190-000, Brazil; 2 Faculdade de Ciências do Desporto e Educação Física, Universidade de Coimbra, 3040-256 Coimbra, Portugal; jpferreira@fcdef.uc.pt (J.P.F.); rodsimendes@gmail.com (R.M.); 3 Polytechnic Institute of Coimbra, Coimbra Education School, Rua Dom João III—Solum, 3030-329 Coimbra, Portugal; goncalodias1976@sapo.pt (G.D.); rmendes@esec.pt (R.M.); 4 SPRINT Sport Physical Activity and Health Research & Innovation Center, 3030-329 Coimbra, Portugal; 5 Laboratório RoboCorp, IIA, Instituto Politécnico de Coimbra, 3040-256 Coimbra, Portugal; 6 CIDAF (UID/DTP/04213/2020), Universidade de Coimbra, 3040-256 Coimbra, Portugal; 7 ESEC-UNICID-ASSERT, Instituto Politécnico de Coimbra, 3040-256 Coimbra, Portugal

**Keywords:** performance, sports, psychology, awareness, non-judgment, refocusing, tactical intelligence, perception

## Abstract

This study aimed to analyze the effect of a flow training program based on mindfulness applied to young and adult elite handball athletes. A quantitative, quasi-experimental, descriptive data analysis approach was carried out. The sample included 105 athletes (51 female and 54 male). The athletes were divided into two groups: (i) experimental (n = 53) and (ii) control (n = 52). The results of the repeated ANOVA indicated that the experimental group achieved significant improvements compared to the control group in decision making (n^2^*p* = 0.086), mental well-being (n^2^*p* = 0.045), dispositional flow state (n^2^*p* = 0.103), non-judgment (n^2^*p* = 0.040), refocusing (n^2^*p* = 0.052), and mindful traits in daily life (n^2^*p* = 0.058). A Pearson analysis demonstrated a positive correlation between dispositional flow state and mindfulness in sport and mental well-being. The correlation analysis also showed an inverse correlation between decision making and sense of control and mindful traits in daily life. The findings revealed that the program can be effective in decision making, mental well-being, dispositional flow state, mindfulness in sport, and mindful traits in daily lives of athletes.

## 1. Introduction

Research into sports performance has increasingly highlighted the fundamental role of the psychological dimension in performance [1,2]. In this sense, Castro Sanchez et al. [3] and Ring et al. [4] indicated that, besides the fact that psychological factors are capable of strongly affecting athletes’ behavior, their own performances can be significantly attributed to the cognitive and emotional changes they undergo. Thus, the trend among current investigation showed the need for developing an optimal mental well-being state among athletes, which would optimize and underlie their performance.

In relation to mental well-being, one of these factors, perhaps the most prominent and desired optimal mental state, is the flow state [5,6]. The flow state has been conceptualized as an optimal mental state of deep concentration and attention [7]. Also, it has been correlated with increased productivity and improved performance [8,9], with the involvement of positive emotions, happiness, and well-being [10,11], and with an adequate direction of attention to the task at hand, without the need for effort [12]. One of the estimates is that by being in flow, athletes are able, even under pressure, to expand their mental state beyond a state of tension, anxiety, and the need for self-control [13].

The search for this optimal mental state has directed research not only towards verifying the factors that influence and correlate with the flow state and its effects on athletes’ behavior, but also towards intervention strategies that might induce it.

Previous research has suggested that mental training based on mindfulness may be associated as a predictor–facilitator for the athlete to improve their dispositional flow state (as a latent projective trait) and achieve the flow state in action (flow experience) [14,15,16]. Besides that, these interventions based on mindfulness have been used as mental training that not only brings emotional–psychological well-being to athletes [17], but also allows for, among other possibilities, a reduction in the risk of injuries [18], an increase in cognitive and emotional flexibility [19,20], a decrease in stress and anxiety levels, a decrease in negative thoughts and self-criticism [21,22], and the possibility of coping with challenging situations [23,24].

A widely accepted concept of mindfulness describes the quality of paying attention to the present moment, in an intentional manner, with curiosity and openness, and without judgment [25]. Mindfulness involves the condition of consciously relating to one’s own feelings, emotions, and thoughts, without resistance or experiential avoidance [26,27,28].

Acknowledging that an athlete’s high performance is associated with meditation in movement [29], some authors have sought to highlight the possible correlations between mindfulness and flow. Hence, Carraça et al. [30] demonstrate that there is a possible experiential compatibility between them, since both are not only associated with an optimal mental state, but mainly because they work in favor of regulated activity. This differs from the wandering state of mind, since no intentional activity occurs in this condition. Schutte and Malouff [15] underscored the extent to which high levels of mindfulness are associated with higher levels of flow.

Gardner and Moore [31] indicated that there is an overlap between the constructs involving flow and mindfulness. For example, knowing that attention, as a form of meta-cognition, plays a regulatory role both in the flow state, by bringing order to consciousness, and in mindfulness, by allowing reality to be presented where attention is directed to, it is possible to point out an experiential compatibility between both, mainly because they work in favor of regulated activity [30,32]. Jackson and Csikszentmihalyi [6] point out that one of the ways to enter flow is to focus on the present moment.

Broadly, this study is justified by the comprehension that flow training based on mindfulness not only improves athletes’ mental well-being but develops their awareness in the present moment. Besides that, it influences athletes to establish a relationship of acceptance, non-judgement, and defusion with their most perturbing malfunctioning thoughts [2]. Training can also lead them to psychological flourishing, as it induces self-compassion, sports assertiveness, and emotional self-regulation. This would provoke a decrease in reactivity in favor of an intentional action [33,34]. 

This context becomes relevant once there is a need for an investigation that clarifies the advantages of the flow state, mainly regarding the cognitive and emotional processes that support the athlete’s performance [35]. One of these cognitive processes is decision making. As far as we know, there are still few studies that can analyze flow state along with tactical decision-making processes in team sports (e.g., handball), especially when analyzing its effects on the tactical elements of performance [16]. In this way, the present study offered a training program that aimed to improve the quality of tactical decision-making processes, and related this quality to mental well-being and an improvement in mindfulness in sports and daily life contexts. 

Therefore, the main goal of this study was to analyze whether a flow training program based on mindfulness had an effect on dispositional flow state (DFS), mental well-being (MWB), mindful traits in daily life (MAAS), mindfulness in sport (MIS), and decision making (DM) in handball athletes. Our research analyzed the following hypotheses: H1—the flow training program based on mindfulness has an impact on dispositional flow state, decision making, mental well-being, mindfulness in sport, and mindful traits in daily life; and H2—the dispositional flow state and mindfulness in sport positively correlate with mental well-being, mindful traits in daily life, and decision making.

Taking these hypotheses into consideration, the variables of interest were tactical decision-making processes; mental well-being; mindfulness in sports and in daily life; and the dispositional flow state. Besides that, the presentation of the two hypotheses became relevant not only to test the impact of the training program over these selected variables, but also as a condition to investigate the extent to which flow and mindfulness may, indeed, influence the elements that affect an athlete’s performance, or more specifically, decision making, or sports performance itself. In other words, it was intended to verify the strength of the relationships of flow and mindfulness with other variables.

## 2. Materials and Methods

### 2.1. Methods

This research is characterized as quantitative, as it used a quasi-experimental, non-randomized research method, pre- and post-tests, and descriptive data analysis.

### 2.2. Sample

The sample included 105 athletes, 51 female and 54 male. Furthermore, 58 athletes belonged to the adult category and 47 to the youth category. The participants were divided into an experimental group (n = 53) and a control group (n = 52). The athletes were directed to the experimental or control group according to their team/club performance in a Brazilian context. So, the experimental group was composed of athletes who belonged to clubs/teams that participated in national competitions. The control group was formed by athletes who belonged to clubs/teams that played regional-level competitions. The composition of the groups by sex (female and male), category (youth: U19 and adult: 20+), average age, years of practice, and weekly hours of training were distributed, according to Table 1.

The inclusion criteria for the study were the following: (i) the athlete was actively training in a club/institution; (ii) he or she was in the youth or adult category; and (iii) he or she was available to follow the evaluation protocol (for the control group) or the intervention program based on mindfulness combined with the evaluation protocol (for the experimental group).

The exclusion criteria were the following: (i) the non-signing of the informed consent form by the athlete (in this case, athletes over 18 years old) or by their parents or guardians (in this case, athletes under 18); (ii) athletes who were undergoing drug treatment regarding psychological disorders; and (iii) athletes who had already participated in a meditation practice protocol or program. Athletes in the control group were offered the possibility of participating in the intervention program based on mindfulness after completing all stages of the research [6,36,37].

### 2.3. Instruments

We used a sociodemographic questionnaire (SDQ), prepared for the purposes of this research. This questionnaire contained general identification data (age, gender, and level of education). It also posed closed questions that met the inclusion/exclusion criteria (e.g., how long have you been practicing this sport? How many hours of training do you complete per week? Are you on any type of drug treatment for psychological disorders? Have you ever participated in or are you participating in any meditation practices?).

The mindfulness inventory for sport (MIS-original) [38], the Brazilian version [39], was also used (reliability was ensured by using the Brazilian version: awareness α = 0.75; non-judgement α = 0.80; and refocusing α = 0.69). For this study, pre-intervention was as follows: awareness α = 0.77, non-judgement α = 0.842, and refocusing α = 0.678; post-intervention was as follows: awareness α= 0.800, non-judgement α = 0.836, and refocusing α = 0.702). The inventory was used to assess the quality of athletes’ mindfulness. It consists of 15 items, equally distributed in 3 subscales that can be used to assess the following factors: awareness; non-judgment; and refocusing. Each item is answered using a 6-point Likert scale limited by the following extremes: 1 = not at all, and 6 = very much.

The tactical intelligence test in handball [40,41] was also conducted (Content Validity (CVC ≥ 0.80), inter-observer agreement (CEO ≥ 80%), and Reproducibility (R = *p* ≤ 0.09). The declarative tactical knowledge level protocol verifies the levels of the cognitive processes of handball athletes, which comprise the level of perception and tactical decision-making processes in game-problem situations through scenes recorded on video. There are 11 scenes of a handball game, with each scene lasting an average of 8 to 10 s and focusing on the offensive situations of the attacking player with the ball. It was necessary to make the decision to pass, feint, or shoot in each of these scenes. There was a total correctness template, and the athlete received a respective classification score.

A dispositional flow state (DFS) measure [42], the Brazilian version [43], was used (reliability was ensured by using the Brazilian version: α > 0.90). For this study, pre-intervention α = 0.913 and post-intervention α = 0.935. This instrument is used to assess an athlete’s perception of several indicators of predisposition to the flow state. This version consisted of 36 items representing the nine dimensions of flow: (i) challenge–skill balance (CSB), (ii) action–awareness merging (AAM), (iii) clear goals (CGs), (iv) unambiguous feedback (UF), (v) total concentration (TC), (vi) sense of control (SC), (vii) loss of self-consciousness (LSC), (viii) transformation of time (TT), and (ix) autotelic experience (AE), in addition to total dispositional flow state (DFS_total_). Each item was answered on a 5-point Likert scale (1—never; 5—always).

The Warwick–Edinburgh mental well-being scale (WEMWBS—Brazilian Version) [44] was used (reliability was ensured from the use of the Brazilian version: α = 0.89). For this study, pre-intervention: α = 0.939 and post-intervention α = 0.943. This is a scale that allows for the measurement of the mental well-being of the general population in a unidimensional way and is composed of 14 items measured on a 5-point Likert scale (1 = never; 5 = always).

The Mindful Attention Awareness Scale (MAAS) [45], the Brazilian version [46], was used (reliability was ensured by using the Brazilian version: α = 0.83). For this study, pre-intervention: α = 0.874 and post-intervention α = 0.870. This scale was used to measure people’s tendency to be fully attentive in daily life, that is, how much internal and external dispositions are fully perceived in the present moment. It is a scale with a single general dimension composed of 15 items, with each item being answered on a 6-point Likert scale (1 = almost always; 6 = almost never).

### 2.4. Procedures

The period of recruitment for athletes began in September 2022. For the experimental and control groups (both male and female), the period of recruitment and pre-intervention assessments took place between the 6th and 21st of September, and post-intervention assessments took place between 25 November and 12 December 2022. The nine-week intervention program took place between 22 September and 24 November 2022. Both pre-intervention and post-intervention assessments happened face to face, in a room offered by the club/institution. The assessment instruments, as well as the test administrator, were always the same. In both contexts (pre- and post-intervention), the athletes individually filled out the forms related to the instruments using an individual device. 

The athletes who participated in this study were recruited, firstly by invitation and consent from the club/institution where they play. Secondly, a meeting, with all the athletes who were interested, was conducted to clarify aspects regarding the research. Finally, each participant demonstrated a voluntary acceptance of their participation.

### 2.5. Study Design

**Figure 1:**
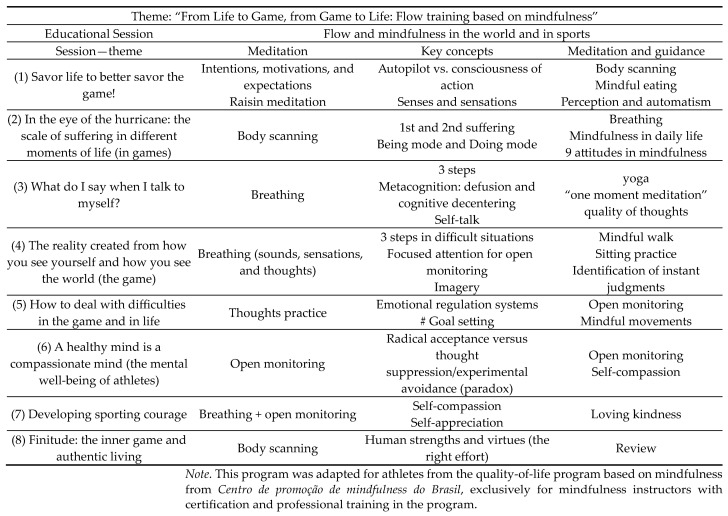
Flow training intervention program based on mindfulness.

The experimental groups (male adult, female adult, male youth, and female youth) participated individually in the nine-week mindfulness intervention program (Figure 1), with the first week providing clarification on how the program worked and education about flow (adapted) [47] and mindfulness [48,49]. 

The following eight weeks consisted of the application of the training program itself. This program followed all MBI-TAC recommendations and guidelines for what a mindfulness intervention program should contain [50,51], thus guaranteeing at least 30 min per session of exclusive meditation and inquiry practices, as well as the provision of meditation exercises at home.

The sessions also contained psycho-educational exercises, with the aim of improving sporting performance, developing psychological skills and their effects, and leading exercises about compassion, acceptance, and commitment [30,37,52,53,54], and their relationships with mental well-being and flow. The sessions occurred face to face, once a week for each experimental group individually, and in a room offered by the club/institution, and lasted between 90 and 120 min. All sessions, for all the intervention groups, were carried out by the same instructor, who is the main author of this article. Besides possessing an instructor certificate for mindfulness-based interventions, granted by Centro de Promoção de Mindfulness do Brasil, he also specializes, and has extensive experience, in sports training. All home meditation sessions were directed according to the mindfulness-based stress reduction program [25,48] and the MBSoccer program in a sports context [37,52,55]. 

### 2.6. Data Analysis

Statistical analyses were performed using the JAMOVI program (2022 version 2.3). After controlling and correcting non-responses to each one of the items (per protocol sample—missing data analysis) and excluded participants (n = 15) that were not part of final sample, the repeated *t*-test was applied to verify the assumptions of normality and homogeneity on the pre- and post-intervention measures and the independent *t*-test to verify significant differences between groups in relation to the variables’ dispositional flow state and their respective dimensions. These included mindfulness applied to sport and its respective factors, mindful traits in daily life, decision making, and mental well-being before intervention.

ANOVA tests for repeated measures were performed to examine intragroup and intergroup differences (experimental versus control) pre- and post-intervention, with post hoc analysis conducted using Scheffe corrections when necessary. This presented, in repeated ANOVA measures, the effect size in partial eta squared (n^2^*p*). For interpretation purposes, we adopted the following reference values: n^2^*p* = 0.0099 (small effect), n^2^*p* = 0.0588 (medium effect), and n^2^*p* = 0.1379 (large effect) [56]. Pearson’s correlation was also applied to verify the strength of the relation between the pre- and post-intervention variables in the experimental group. The following reference values were adopted to interpret the data: r = 0.10 (small effect), r = 0.30 (medium effect), and r = 0.50 (large effect) [57].

### 2.7. Ethical Considerations

This study was submitted and approved under number 5.625.093 by the Ethics Committee of the *Instituto Federal do Rio Grande do Norte* (IFRN—Brasil). All participants (athletes) were invited to participate anonymously, confidentially, and voluntarily in the study. Everyone received explanations about the objectives of the study and read and signed the informed consent form in accordance with CNS resolution no466/12. This study does not foresee any risk or harm to the participants. 

## 3. Results

According to the Kolmogorov–Smirnov normality test, the variables decision making, challenge–skill balance, and autotelic experience presented a non-normal distribution (*p* < 0.05, respectively). Using Levene’s homogeneity test, it was found that all research variables (dispositional flow state and its respective dimensions, mindfulness applied to sport and its respective factors, mental well-being, mindful traits in daily life, and decision making) are homogeneous (*p* > 0.05). In the independent *t*-test, the non-parametric Mann–Whitney test was adopted for the variables decision making, challenge–skill balance, and autotelic experience.

The independent *t*-test identified that there were significant differences between the groups (control versus experimental) in the pre-test, but only in the internal dimensions of dispositional flow state (challenge–skill balance—CSB (U = 962, *p* < 0.016), clear goals—CGs (t (101) = −3.314, *p* < 0.001), and unambiguous feedback—UF (t (101) = −2.233, *p* < 0.028).

The results of the ANOVA repeated measures obtained statistically significant differences between the groups (control versus experimental) in relation to some of the measures (Table 2). These measures represent mean scores in the pre-intervention test and post-intervention retest, and the difference between them. A significant effect of the intervention emerged in relation to decision making (DM), with a medium to large effect size. The experimental group obtained a statistically significant mean difference in accuracy in decision making when compared to the control group. In the post hoc analysis, significant differences were found in the group*gender interaction. The female experimental group had a significantly higher mean accuracy rate compared to the female control group (M = −1.122; SD = 0.260; p_scheffe_ < 0.001).

Regarding dispositional flow state, there were significant differences between the groups in five dimensions, plus the total dispositional flow state. The challenge–skill balance (CSB) dimension showed significant differences between the groups with a large effect size. Thus, on average, the experimental group was able to better perceive and balance the challenge required by the task in relation to the skills they had available. The dimension clear goals (CGs) also showed significant differences between the groups with a large effect size. In this way, the experimental group managed, on average, to have more clarity about the objectives to be achieved in relation to the control group.

Unambiguous feedback was another dimension that showed significant differences between groups with a large effect size. The experimental group tended to understand more clearly the feedback received about their performance. In post hoc analyses, significant differences in the group*category interaction could also be seen. The youth experimental group tends to have a better understanding, on average, of the feedback received than the adult control (M = −0.483; SD = 0.162; p_scheffe_ < 0.035) and youth control (M = −0.845; SD = 0.172; p_scheffe_ < 0.001). The data revealed that the adult experimental group managed to have, on average, better knowledge about the feedback they receive about their performance in relation to the youth control group (M = −0.659; SD = 0.166; p_scheffe_ < 0.002).

Total concentration on the task (TC) also showed statistically significant differences between groups, with a medium effect size. Accordingly, the experimental group, on average, increased its ability to concentrate on the tasks to be performed compared to the control group. Sense of control (SC) was another dimension that showed statistically significant differences between the groups, with a medium to large effect size. The experimental group, on average, tends to believe that they have better control over the situation and feel more confident compared to the control group.

The experimental group showed, on average, a better total dispositional flow state (DFS_total_), with a medium to large effect size, in relation to the control group. In post hoc analyses, there were significant differences in the group*category interaction. The youth experimental group tends to have a better disposition, on average, for the flow state in relation to the youth control group (M = −0.476; SD; 0.124; p_scheffe_ < 0.003).

Regarding the mindfulness inventory for sport (MIS), the ANOVA results indicated statistically significant differences between groups, with medium effect size on the non-judgment factor (MIS-NJ). The control group, on average, obtained higher applications of non-judgment in relation to the experimental group.

The refocusing factor (MIS-RE) also showed statistically significant differences between the groups, with a medium effect size. Thus, on average, the experimental group was better able to reperceive their experiences in the present moment, better than the control group.

Regarding mental well-being (MWB), the ANOVA analysis showed significant differences with a medium effect size. On average, the intervention improves the mental well-being of athletes in the experimental group compared to the control group.

In relation to athletes’ mindful traits in daily life (MAAS), there was also a significant difference with a medium effect size. So, the athletes in the experimental group showed improved mindfulness in different moments in their daily lives.

To analyze the strengths of the relationships between the evaluated constructs, Pearson’s correlation was applied between the pre- (a) and post-intervention (p) measures (Table 3). 

The total dispositional flow state (DFSt_a_), in addition to presenting the expected correlations between its own internal dimensions, presented medium to large positive correlations with awareness (AW_p_) (r = 0.499, *p* < 0.001, 95%CI [0.261–0.679]), medium positive correlations with refocusing (RE_p_) (r = 0.296, *p* < 0.05, 95%CI [0.025–0.527]), and medium to large positive correlations with mental well-being (MWB_p_) (r = 0.349, *p* < 0.05, 95%CI [0.085–0.568]). Awareness, which is a factor of mindfulness in sport (MIS-AW), obtained a medium positive correlation with the internal dimensions of the dispositional flow state autotelic experience (AE_p_) (r = 0.298, *p* < 0.05, 95%CI [0.027–0.528]). 

Refocusing, another factor of mindfulness in sport (MIS-RE_a_), also showed medium positive correlations with total concentration (TC_p_) (r = 0.296, *p* < 0.05, 95%CI [0.026–0.527]), and medium positive correlations with loss of self-consciousness (LSC_p_) (r = 0.341, *p* < 0.05, 95%CI [0.075–0.562]). Furthermore, MIS-RE_a_ presented medium positive correlations with mental well-being (MWB_p_) (r = 0.296, *p* < 0.05, 95%CI [0.025–0.526] and medium positive correlations with mindful traits in daily life (MAAS_p_) (r = 0.324, *p* < 0.05, 95%CI [0.056–0.548]).

Sense of control (SC_a_), one of the dimensions of a dispositional flow state, presented medium negative correlations with decision making (DM_p_) (r = −0.281, *p* < 0.05, 95%CI [−0.514–0.009]). Also, mindful traits in daily life (MAAS_a_) presented medium to large negative correlations with decision making (DM_p_) (r = −0.416, *p* < 0.01, 95%CI [−0.619–0.161]). 

## 4. Discussion

The flow training program based on mindfulness proved to be effective in improving the decision-making processes of athletes in the experimental group in relation to the control group, pre- and post-intervention. There were also significant results on dispositional flow state in the factors non-judgment and refocusing mindfulness in sport, mental well-being, and mindful traits in daily life, confirming the first hypothesis. Besides that, studies have found that there is a relationship between increased flow and mental well-being, achieved through mindfulness training, pre- and post-intervention, and mental resistance, coping strategies, and psychological flexibility [20,58,59].

Hypotheses 2 was partially confirmed. The analysis of the correlations between the measures showed that flow and mindfulness (AW, RE) are interrelated, and they converge and correlate positively with the athlete’s mental well-being. Schutte and Malouff [15] indicated not only a strong association between mindfulness and flow, but also a connection with a variety of beneficial results for practitioners. Therefore, the improvement in mental well-being may not only be accompanied by an excellent disposition for flow and an adequate level of mindfulness, but it may also be an excellent indicator of improved athlete performance [9,47,60]. Positive correlations also indicated that the program may offer the athletes the ability to experiment and increase their dispositional flow state and mindfulness in activities other than those related to sports contexts. 

One of the theoretical understandings of the program established a relationship between the game context and one’s personal life. The program assumed that both are not dissociated and that, therefore, the mental state that the athletes experience on the court may be related to their behavior outside of it. And this has implications for how they feel, how they direct their decisions, and how this reflects on their behaviors. Considering a wider context, the associations between dispositional flow state (DFS), mindfulness in sport (MIS), and mental well-being (MWB) guide us to this analysis [17,37]. 

Mindfulness not only offers athletes the possibility of making a reperception of their own reality, but also awareness of the present moment and a reorientation of actions, performed to break certain response patterns [61]. It is in this sense that the relationship between dispositional flow state and the factors of awareness and refocusing was expected and confirmed. This result indicated that dispositional flow state, awareness, and refocusing may occupy a more important role in an athlete’s performance. 

Thus, these results point to other possible variables that were not tested and that could explain the emergence of this correlation. Perhaps psychological flexibility itself [52], attention [62], or even the role of memory [63] or self-criticism itself [21,22] are factors influencing performance.

Besides that, the relation among these three variables may even indicate that they work in favor of regulated activity and for the benefit of the athlete’s better internal functioning [8,9,32]. This may be related to the fact that high levels of mindfulness are associated with high levels of flow, mainly dispositional flow [15]. However, there is a need for more exploratory studies for a better understanding of this relationship.

When it comes to the direct elements of performance, it is significant to highlight the variables that correlate with decision making. The first one refers to an inverse relation between mindful traits in daily life (MAAS) and decision making (DM). This result suggested that, if the goal is to improve the quality of decision making during the games, it is necessary for the athletes to dissociate their cognitive attention away from external elements [35]. Thus, the athletes themselves may take more functional decisions in response to the problem situations that emerge throughout the match. 

Otherwise, the inverse relation between sense of control (SC) and decision making (DM) was unexpected. It was expected that the athletes, when feeling they have more control over the moment of the match, would make better decisions. This idea implies that the sense of control is present when the athletes believe they have the necessary abilities to perform the action [11,13]. However, the results showed the opposite. The fact that the athletes try to achieve control over unpredictable situations, which is the nature of team sports, may lead them not to make the better decision, but to experience dysfunctional internal states, like anxiety or a loss of self-confidence [19]. These situations, which may be characterized as intimidating, may trigger internal conflicts that lead them to make mistakes and prevent them from achieving excellent performance [22].

Another relevant aspect lies in the fact that the athletes may act automatically or with mindfulness (with curiosity, openness, and non-judgment). While athletes need to understand the problem situation of the game to then make a decision, they also need to accept the fact that they do not control it. This would make them able to self-regulate their response, so as not to react automatically. In general, people who are able to act non-automatically are the ones who can better self-regulate their responses, present a better disposition for mindfulness, and tend not to make judgements [64]. The search for this sense of control may lead the athletes to a certain mental inflexibility, lack of creativity, and originality, which are important aspects of decision making [65]. However, more exploratory studies are necessary to better understand this phenomenon.

It may be considered that, when feeling less need for control over the situations, the athletes may achieve a better internal balance and a better flow of thoughts, which could lead to a better perception of reality. If the fact of perceiving and better thinking improves the choices that are made and the decisions that are taken on the court, then it is necessary to understand how to improve the quality of the relationship between one’s sense of control and the role of perception and thoughts in decision-making in sports contexts. This is because players who understand and think better tend to be more decisive when playing.

As for practical implications, the positive impact of the flow training program based on mindfulness on athletes’ performance and daily life offers important evidence that the program has the potential to be replicated for this population. The results show positive effects on athletes’ mental well-being, decision-making, and mindfulness in sports and in daily life. These positive results indicate that the training shares important features with holistic approaches, which impact athletes’ behavior in a broad perspective. Thus, the training may affect athletes’ self-regulatory processes, which means the recognition, acceptance, and non-judgment of their limiting beliefs, dysfunctional feelings, thoughts, and behaviors. In this way, flow training may become a possibility condition that aids sports performance. 

Although the results of this investigation were promising, there are some limitations to be considered in future investigations. The first limitation reflects the nature of the research methods. This investigation followed a non-randomized design and sample selection, which led to the results here obtained. A suggestion for a future investigation would be a randomized sample and design, which could lead to different results. Another possibility for future investigation lies in increasing the sample size per category. One suggestion is to apply the program with only one category and gender, but with a larger sample. Another suggestion is to apply the program with athletes from other team sports (e.g., basketball or football) and evaluate the effects.

Even though the results regarding decision making are successful, it is important to highlight that the test has its limitations in the transposition to real-life contexts. In fact, other factors involving decision making were not measured. Here, we mention the time it takes the athletes to make the decision, their reaction time, their memory, and their anticipation. Finally, a future research suggestion involves associating training based on mindfulness with the athletes’ creativity, originality, and fluency in decision making, based on an observational flow scale, or even to relate this to mental well-being as a predictor of decision making and autotelic experiences.

## 5. Conclusions

The results of this study suggested that the flow training program based on mindfulness had an effect on developing mindful traits in daily life and factors associated with mindfulness in sport, as well as an improvement in the dispositional flow state and mental well-being, and in the decision making of athletes, confirming Hypothesis 1.

One the other hand, both the dispositional flow state and the mindfulness in sport factors, including awareness and refocusing, acted in an integrated way and correlated with mental well-being, partially confirming Hypothesis 2. In any case, the program also seemed to function as a link between the athletes’ off-court lives and their behavior when playing.

Another point to highlight is the inverse correlations between decision making and sense of control and mindful traits in daily life. This note demonstrates the extent to which the relationship between the flexibility, quality, and flow of thoughts can influence the quality of decision making made by athletes in games. 

In this study, it was not fully confirmed that the dispositional flow state and mindfulness in sport are related to decision making. This relation was observed only in one of the dimensions of the dispositional flow state and mindful traits in daily life. In an overview, this indicates that other factors may be involved. In any case, both flow and mindfulness need to be further studied to better clarify the roles they play in decision making. Finally, it may be the case that the relation between flow and decision making is not a matter of disposition (a latent personality trait) or projection, but of direct experience (experiential flow).

## Figures and Tables

**Table 1:** Group characteristics, according to their categories, sex, average ages, years of practice, and hours of training.

Group	Category	Sex	n	Age		Years of Practice		Hours of Training	
				Average	SD	Average	SD	Average	SD
Control	Adult	Female	14	23.85	4.67	5.71	5.65	3.50	1.78
		Male	15	26.67	8.41	8.90	6.68	4.07	2.63
	Youth	Female	10	17.00	0.81	1.16	0.89	4.10	1.59
		Male	13	17.62	0.65	1.26	0.94	4.00	2.00
Experimental	Adult	Female	16	23.25	2.86	9.19	3.86	8.50	4.08
		Male	13	21.38	1.80	8.23	3.08	11.69	3.54
	Youth	Female	11	17.36	0.67	3.91	1.75	7.64	5.10
		Male	13	17.69	0.48	5.35	1.37	5.62	2.14
General			105	20.99	5.12	5.79	4.78	6.17	4.05

**Table 2:** Mean and standard deviation of pre-and post-intervention plus significant differences and effect sizes on the measures between control and experimental groups.

Measures	Pre-Test		Post-Test		Repeated Anova	Difference (Pre-/Post-Test) (Control versus Experimental)			
	M	SD	M	SD	F	M	SD	*p*-Value	n²*p*
DM	0.055	0.229	−1.098	0.256 *	9.09 (1.97)	−0.543	0.180	0.003 *	0.086
CSB	−0.365	0.151 *	−0.731	0.156 *	27.35 (1.94)	−0.608	0.116	<0.001 *	0.225
AAM	0.094	0.134	−0.025	0.158	0.12 (1.94)	−0.040	0.115	0.728	0.001
CGs	−0.492	0.149 *	−0.544	0.148 *	19.02 (1.94)	−0.543	0.125	<0.001 *	0.168
UF	−0.324	0.145 *	−0.686	0.153 *	24.27(1.94)	−0.571	0.116	<0.001 *	0.205
TC	−0.144	0.158	−0.315	0.152 *	4.34 (1.94)	−0.275	0.132	0.040 *	0.044
SC	−0.167	0.149	−0.568	0.154 *	11.57 (1.94)	−0.416	0.122	<0.001 *	0.110
LSC	−0.173	0.183	−0.114	0.176	2.24 (1.94)	−0.195	0.130	0.138	0.023
TT	0.184	0.164	0.260	0.165	1.59 (1.94)	0.178	0.141	0.209	0.017
AE	0.285	0.151	−0.211	0.131	0.02 (1.94)	−0.018	0.115	0.874	0.000
DFS-total	−0.124	0.105	−0.325	0.107 *	10.84 (1.94)	−0.277	0.084	0.001 *	0.103
MIS-AW	−0.038	0.176	−0.304	0.172	1.99 (1.96)	−0.208	0.147	0.161	0.020
MIS-NJ	0.407	0.222	0.286	0.232	4.01 (1.96)	0.387	0.193	0.048 *	0.040
MIS-RE	−0.076	0.164	−0.441	0.165 *	5.30 (1.96)	−0.309	0.134	0.023 *	0.052
MWB	−0.147	0.157	−0.416	0.160 *	4.54 (1.96)	−0.297	0.139	0.036 *	0.045
MAAS	−0.298	0.183	−0.473	0.169 *	5.86 (1.96)	−0.377	0.156	0.017 *	0.058

***Note*.** M—mean; SD—standard deviation; DM—decision making; CSB—challenge–skill balance; AAM—action–awareness merging; CGs—clear goals; UF—unambiguous feedback; TC—total concentration on the task; SC—sense of control; LSC—loss of self-consciousness; TT—transformation of time; AE—autotelic experience; DFS-total—total dispositional flow state; MIS-AW—awareness; MIS-NJ—non-judgment; MIS-RE—refocusing; MWB—mental well-being; and MAAS—mindful traits in daily life; * (*p* < 0.05).

**Table 3:** Correlation matrix between decision making, dispositional flow state and its fundamentals, awareness, non-judgment, refocusing, mental well-being, and mindful traits in daily life, pre- (a) and post (p)-intervention.

	DMa	CSBa	AAMa	CGa	UFa	TCa	SCa	LSCa	TTa	AEa	DFSa	AWa	NJa	REa	MWBa	MAASa
DMp	0.07	−0.20	0.08	−0.17	−0.12	−0.08	−0.28 *	0.00	0.24	−0.23	−0.12	−0.20	−0.18	0.02	0.03	−0.41 *
CSBp	−0.14	0.35 *	0.28 *	0.22	0.39 *	0.39 *	0.40 *	0.18	0.20	0.46 *	0.46 *	0.16	0.10	0.17	0.49 *	0.23
AAMp	−0.20	0.08	0.25	−0.14	0.10	0.10	0.02	0.11	0.08	0.11	0.11	−0.16	−0.24	−0.12	0.06	−0.22
CGp	0.02	0.17	0.03	0.37 *	0.32 *	0.36 *	0.46 *	−0.09	0.03	0.13	0.27	0.06	−0.04	0.15	0.19	0.23
UFp	−0.00	0.31 *	0.24	0.32 *	0.48 *	0.31	0.33 *	0.12	0.21	0.53 *	0.45 *	0.05	0.03	0.06	0.42 *	0.16
TCp	−0.00	0.33 *	0.28 *	0.28 *	0.31 *	0.31 *	0.35 *	0.03	0.30 *	0.26	0.38 *	0.08	−0.07	0.29 *	0.31 *	0.14
SCp	0.07	0.30 *	0.22	0.30	0.39 *	0.33 *	0.38 *	0.08	0.04	0.32 *	0.37 *	0.15	−0.00	0.25	0.37 *	0.20
LSCp	−0.06	0.08	0.22	0.11	0.12	0.05	0.26	0.05	0.20	0.02	0.15	−0.08	−0.11	0.34 *	0.06	0.19
TTp	0.03	−0.09	0.17	−0.04	−0.00	−0.02	0.01	0.00	0.54 *	−0.06	0.07	−0.00	−0.17	−0.01	−0.23	−0.16
AEp	−0.01	0.36 *	0.28 *	0.16	0.38 *	0.39 *	0.15	0.28 *	0.35 *	0.39 *	0.45 *	0.29 *	−0.25	0.26	0.27	−0.00
DFSp	−0.05	0.30 *	0.32 *	0.25	0.40 *	0.35 *	0.38 *	0.10	0.32 *	0.35 *	0.44 *	0.08	−0.12	0.23	0.31 *	0.12
AWp	0.16	0.24	0.25	0.46 *	0.44 *	0.32 *	0.38 *	0.20	0.54 *	0.31 *	0.49 *	0.39 *	−0.17	0.36 *	0.33 *	0.13
NJp	−0.07	−0.21	−0.13	−0.13	−0.31 *	−0.17	−0.14	−0.09	−0.29 *	−0.19	−0.26	−0.31	0.42 *	−0.24	−0.14	0.03
REp	0.11	0.20	0.25	0.25	0.33 *	0.18	0.30 *	0.02	0.20	0.15	0.29 *	0.23	−0.14	0.42 *	0.29 *	0.15
MWBp	0.06	0.17	0.39 *	0.30 *	0.26	0.30 *	0.36 *	0.17	0.23	0.04	0.34 *	0.14	0.02	0.29 *	0.36 *	−0.07
MAASp	−0.026	0.200	0.04	0.24	0.16	0.18	0.37 *	0.07	0.11	0.06	0.22	0.14	0.02	0.32 *	0.22	0.17

***Note*.** DM—decision making;—CSB—challenge–skill balance; AAM—action–awareness merging; CGs—clear goals; UF—unambiguous feedback; TC—total concentration on the task; SC—sense of control; LSC—loss of self-consciousness; TT—transformation of time; AE—autotelic experience; DFS—dispositional flow state; AW—awareness; NJ—non-judgment; RE—refocusing; MWB—mental well-being; and MAAS—mindful traits in daily life. * *p* < 0.05.

## Data Availability

The data presented in this study are available on request from the corresponding author due to privacy and ethical reasons.
